# TAp73 regulates mitochondrial dynamics and multiciliated cell homeostasis through an OPA1 axis

**DOI:** 10.1038/s41419-024-07130-6

**Published:** 2024-11-08

**Authors:** Niall A. Buckley, Andrew Craxton, Xiao-Ming Sun, Emanuele Panatta, Lucia Giraldez Pinon, Sina Beier, Lajos Kalmar, Jaime Llodrá, Nobuhiro Morone, Ivano Amelio, Gerry Melino, L. Miguel Martins, Marion MacFarlane

**Affiliations:** 1 MRC Toxicology Unit, University of Cambridge, Cambridge, UK; 2 Safety Sciences, Clinical Pharmacology and Safety Sciences, BioPharmaceuticals R&D, AstraZeneca, Cambridge, UK; 3 Department of Experimental Medicine, University of Rome “Tor Vergata”, Rome, Italy; 4 Division for Systems Toxicology, Department of Biology, University of Konstanz, Konstanz, Germany

**Keywords:** Mitochondria, Apoptosis, Respiratory tract diseases, Tumour-suppressor proteins

## Abstract

Dysregulated mitochondrial fusion and fission has been implicated in the pathogenesis of numerous diseases. We have identified a novel function of the p53 family protein TAp73 in regulating mitochondrial dynamics. TAp73 regulates the expression of Optic Atrophy 1 (OPA1), a protein responsible for controlling mitochondrial fusion, cristae biogenesis and electron transport chain function. Disruption of this axis results in a fragmented mitochondrial network and an impaired capacity for energy production *via* oxidative phosphorylation. Owing to the role of OPA1 in modulating cytochrome *c* release, TAp73^−/−^ cells display an increased sensitivity to apoptotic cell death, e.g., *via* BH3-mimetics. We additionally show that the TAp73/OPA1 axis has functional relevance in the upper airway, where TAp73 expression is essential for multiciliated cell differentiation and function. Consistently, ciliated epithelial cells of *Trp73*^*−/−*^ (global p73 knock-out) mice display decreased expression of OPA1 and perturbations of the mitochondrial network, which may drive multiciliated cell loss. In support of this, *Trp73* and *OPA1* gene expression is decreased in chronic obstructive pulmonary disease (COPD) patients, a disease characterised by alterations in mitochondrial dynamics. We therefore highlight a potential mechanism involving the loss of p73 in COPD pathogenesis. Our findings also add to the growing body of evidence for growth-promoting roles of TAp73 isoforms.

## Introduction

The transcription factor p73, together with p53 and p63, is a member of the p53 family [1–3]. This family shares a common ancestor and a highly conserved DNA-binding domain, enabling each member to activate a shared set of genes responsible for cell-cycle arrest and apoptosis following DNA damage [4–6]. However, they also have distinct mechanisms of regulation by upstream signalling pathways and post-translational modifications [7, 8]. The *Trp73* gene contains two alternative promoters, giving rise to, respectively, transcriptionally proficient transactivation (TAp73) and anti-apoptotic N-terminally deleted dominant negative (ΔNp73) isoforms [8]. Moreover, further complexity arises from C-terminal alternative splicing, which modulates p73 function [9, 10].

In addition to its p53-like functions, p73 fulfils unique DNA-damage independent roles [11]. Indeed, the expanding set of TAp73 functions in embryonic development, tissue homeostasis and cancer demonstrate its vast functional pleiotropy [12]. The generation of p73 knockout mouse models has uncovered important roles of p73 in development of the nervous system [13], the control of metabolism [14–16], spermatogenesis [17], angiogenesis [18] and multiciliogenesis [19, 20]. However, the molecular underpinnings of the diverse tissue dysfunction require further investigation. One such potential mechanism is a role of TAp73 in the control of metabolism and mitochondrial function [14]. For example, deleting TAp73 from mouse embryonic fibroblasts (MEFs) leads to a downregulation of the respiratory complex IV subunit *Cox4i1*. This results in reduced complex IV activity, and a concomitant decrease in cellular ATP, oxygen consumption, and a premature ageing phenotype in vivo [16]. Additional molecular mechanisms by which TAp73 regulates mitochondrial function and metabolism are through the direct transcriptional control of *GLS2*, the gene encoding glutaminase type 2 [21], glutamine metabolism [22], and the synthesis of serine [23].

We present data highlighting a novel transcriptional role for TAp73 in regulating the mitochondrial shaping protein Optic Atrophy 1 (OPA1) in vitro and in vivo. Functionally, OPA1-dependent mitochondrial fusion supports increased rates of mitochondrial oxidative phosphorylation [24]. Moreover, independently of fusion, OPA1 regulates the assembly of mitochondrial respiratory complexes and OXPHOS efficiency, owing to its function in maintaining cristae morphogenesis [25, 26]. The importance of OPA1 in the maintenance of mitochondrial structure, genome, and function is further evident from mouse and patient models where mutation or loss of OPA1 gives rise to several pathophysiological outcomes including wasting of skeletal muscle and autosomal dominant optic atrophy [27, 28]. We show that ablation of TAp73 in vitro elicited a downregulation of OPA1 expression, fragmentation of the mitochondrial network, impaired bioenergetic function, and increased sensitivity to apoptosis. We therefore present an overarching mechanism by which TAp73 regulates mitochondrial dynamics and respiratory function.

We have also investigated the TAp73/OPA1 axis in vivo, focussing on the airway ciliated epithelium in *Trp73*^*−/−*^ mice, which have a global deletion of all p73 isoforms. Importantly, although the generation of motile cilia is severely abrogated in both *Trp73* and TAp73 knockout mice, TAp73 isoforms are necessary and sufficient for functional multiciliogenesis [19]. Multiciliated cells lacking TAp73 are characterised by short appendages with impaired mucociliary clearance. Multiciliogenesis proceeds *via* an alternative cell cycle to coordinate the formation of motile cilia [29], is a highly energy dependent process, and intricately linked to mitochondrial function [30]. Integral to cilia microtubule polymerisation is the interaction of ATP at the exchangeable GTP site of tubulin, with insufficient ATP generation leading to decreased microtubule stability [31, 32]. Such a phenomenon was evident in OPA1-deficient neutrophils, which produce insufficient ATP for neutrophil extracellular trap formation [33]. As such, the regulation of OPA1 by TAp73 in the ciliated epithelium suggests that TAp73-dependent metabolic regulation may participate in the multiciliogenesis process [14]. In support of this, we show that *Trp73* ablation led to a decrease in OPA1 expression and altered mitochondrial morphology in the ciliated epithelium of the mouse airway. This therefore represents a potential mechanism underpinning multiciliated cell loss in *Trp73* null mice and may also be relevant in chronic obstructive pulmonary disease (COPD) pathogenesis, as we discovered that COPD patient cohorts display decreased *Trp73* and *OPA1* expression. Furthermore, this novel mechanism represents a growth-promoting function of TAp73 isoforms. Indeed, although p53 is lost or mutated in about half of human cancers, the same is not true for p73 [34, 35]. On the contrary, certain p73 isoforms are overexpressed in a range of cancers and influence disease prognosis [36–39]. Overall, we have uncovered a novel mechanism by which TAp73 regulates mitochondrial morphology in vitro and in vivo. Due to the COPD-like pathology of *Trp73*^*−/−*^ mice airways, loss of this axis may therefore contribute to the poorly understood process of COPD pathogenesis [40, 41].

## Results

### TAp73 regulates OPA1 expression and binds with the promoter region

**Fig. 1:**
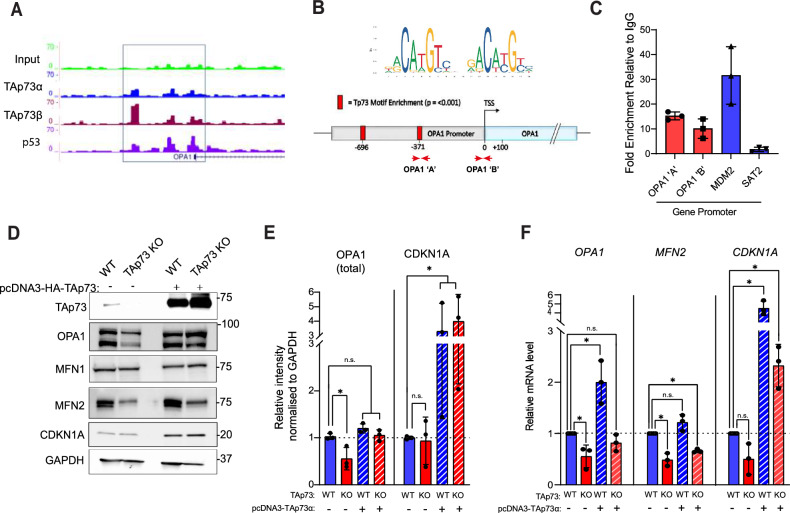
TAp73 regulates the expression of OPA1. **A** Interrogation of ChIP-seq data indicated binding of TAp73α, TAp73β and p53 to the putative OPA1 promoter region. Sequencing read files were obtained from the GEO data set GSE15780, and tracks shown are for the indicated transcription factors at selected genes. **B**, **C** Targeted ChIP of TAp73 bound chromatin. RT-qPCR primers were designed in the promoter region of the OPA1 gene (OPA1 ‘A’ and OPA1 ‘B’). Red squares indicate regions enriched for the Trp73 motif (p < 0.001). qPCR was performed to quantify the fold enrichment of the OPA1 promoter region in an IP sample relative to IgG control. Enrichment of MDM2 and SAT2 promoter regions were assayed as positive and negative controls, respectively. qPCR was carried out on 3 independent ChIP experiments and data shown as individual data points ± SD (n = 3). **D** Representative western blot of mitochondrial fusion proteins in TAp73 KO and WT control. Cells were transfected with either EV or TAp73α expression construct for 24 h. **E** Densitometry analysis of western blot data shown in (D). Band intensity was calculated for total OPA1 (long and short isoforms) and CDKN1A (positive control). Signal intensity was normalised to loading control and reported relative to WT cells transfected with empty vector (n = 3). *P ≤ 0.05, (n.s.) not significant (Student’s t-test, comparison of indicated condition with WT empty vector control). **F** RT-qPCR was performed against *OPA1*, *MFN2* and *CDKN1A* genes and expression values calculated using the ∆∆Ct method, relative to WT empty vector control. Data shown as mean ± SD (n = 3). *P ≤ 0.05, (n.s) not significant (Student’s t-test, comparison of indicated condition with WT empty vector control).

TAp73 has a number of roles in regulating cellular metabolism [42, 43]. We have previously reported a correlation between TAp73 and OPA1 expression in H1299 cells, suggesting that the control of metabolic processes by TAp73 might also occur through the regulation of mitochondrial morphology [9]. To address this possibility, genome-wide binding sites for TAp73α, TAp73β and p53 were interrogated from previously published ChIP-seq data [44]. The binding profile upstream of the OPA1 transcription start site (TSS) showed an enrichment of reads at distinct loci in immunoprecipitated samples, indicating TAp73 binding (Fig. 1a). Moreover, this enrichment was evident for both TAp73α and TAp73β isoforms, suggesting that the SAM domain of TAp73α is not required for this interaction. Furthermore, p53 binding was detected close to the OPA1 transcription start site, suggesting that the ability of TAp73 to bind the OPA1 promoter region may be conserved throughout the p53 family (Fig. 1a, S1a). In addition, ChIP-seq data sets provided the opportunity to explore the possibility that TAp73 may regulate additional genes that influence mitochondrial dynamics. We identified TAp73α, TAp73β and p53 binding at genomic loci for the mitochondrial fusion gene *MFN2* (Fig. S1a), together with fission regulators *MFF* and *FIS1* (Fig. S1b). Contrastingly, TAp73 binding was not evident for the master regulator of mitochondrial fission, *DNM1L* (Fig. S1b), or the OPA1 processing factors *OMA1* and *YME1L*. In addition, GO enrichment analysis of 1769 TAp73 target genes, identified using in-situ mouse tracheal ChIP-seq, identified an overrepresentation of genes that regulate mitochondrial membrane organisation (Gene Ontology ID: 0007006). These included *OPA1*, *MFN2* and *PPARGC1A*, the gene encoding a subunit of PGC-1α (Fig. S1b).

We subsequently selected the OPA1 gene for further investigation due to its important roles in mitochondrial fusion, cristae morphogenesis and respiratory function [24, 25]. Targeted IP of TAp73-bound chromatin followed by qPCR of the OPA1 promoter found an enrichment at two regions upstream of the OPA1 TSS, relative to IgG control (Fig. 1b, c). This showed that overexpressed TAp73α can bind the OPA1 promoter. Moreover, the OPA1 promoter region showed a significant enrichment for the p73 binding motif at two loci (Fig. 1b).

Next, we generated TAp73 knockout (KO) H1299 cell lines using CRISPR/Cas9 targeting to investigate the effect of TAp73 ablation OPA1 expression (Fig. S2). Genetic ablation of TAp73 isoforms resulted in a decrease in OPA1 expression at the protein and mRNA levels (Fig. 1d–f). Moreover, ectopic expression of TAp73 was sufficient to restore OPA1 expression in TAp73 KO cells. To confirm the functional activity of overexpressed TAp73, we also show a potent upregulation of the TAp73 target CDKN1A at the protein and mRNA levels (Fig. 1d–f). Together, these data highlight OPA1 as a downstream transcriptional target of TAp73. We additionally found that the expression of MFN2 was downregulated in TAp73 KO cells (Fig. 1d, f). Taken alongside ChIP-seq data (Fig. S1a), this observation suggests *MFN2* may also be a TAp73 target gene, albeit its expression was not rescued by ectopic expression of TAp73.

### Disruption of the TAp73/OPA1 axis alters mitochondrial morphology and bioenergetics

**Fig. 2:**
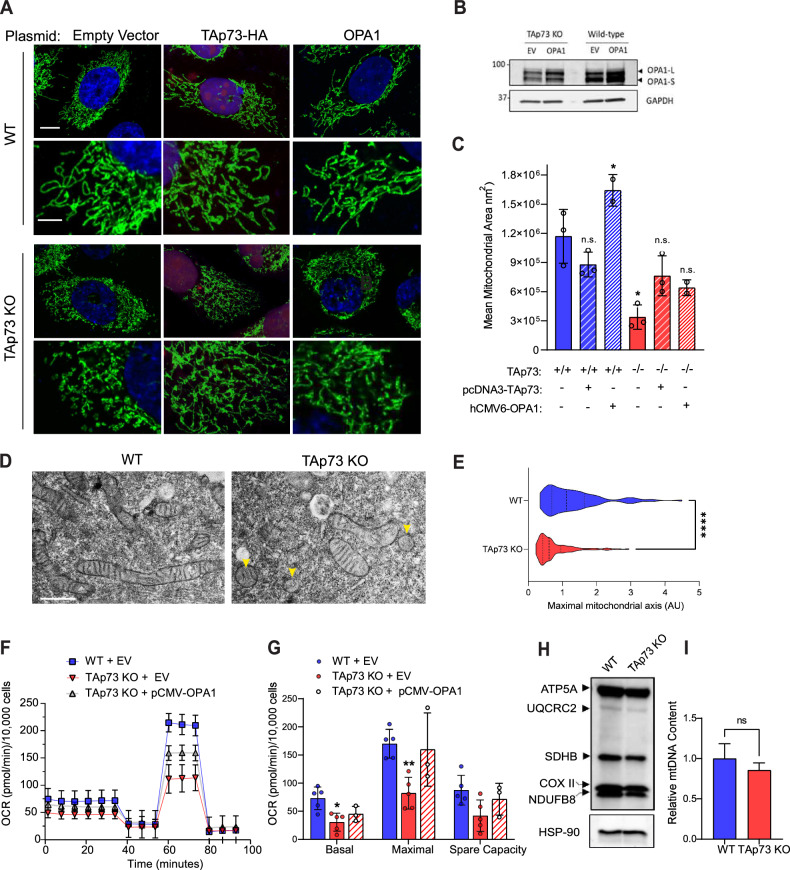
TAp73 KO cells display fragmented mitochondria and impaired ETC function. **A** WT or TAp73 KO H1299 cells were transfected with the indicated plasmids for 24 h and IF carried out against ATP5B (green) with DAPI nuclear counterstain (blue). Cells transfected with HA-TAp73α expression plasmid were stained for HA as a transfection control (red). Lower magnification images, scale bar = 10 μm; Callout images, scale bar = 4 μm. **B** Representative western blot of OPA1 expression following transfection of WT and TAp73 KO cells with pCMW-OPA1 construct. **C** Quantification of mitochondrial morphology from (**A**) using Zeiss Intellesis module, trained to segment individual mitochondria. Statistical significance for each condition was calculated using Student’s t-test, comparing with WT EV control (column 1); *p < 0.05, (ns) not significant (n = 3). **D** Transmission electron micrographs of mitochondrial morphology from WT and TAp73 KO cells. Scale bar = 100 nm. **E** Mitochondrial length measurements obtained from (**D**). ****p < 0.0001 in Student’s t-test. A minimum of 100 mitochondria were measured from n = 3 independent biological replicates. **F**, **G** Mitochondrial stress test performed on Seahorse XFe96 analyser. Canonical mitochondrial inhibitors injected sequentially as labelled (Oligomycin = 2 μM, FCCP = 500 nM, Antimycin A/ Rotenone = 2 μM). The indicated mitochondrial stress test parameters were calculated from OCR data. Data were corrected for non-mitochondrial OCR, normalised to cell number, and are shown as mean ± SD (n = 3). *P ≤ 0.05 and **P ≤ 0.01 in Student’s t-test relative to WT control. **H** Western blot of the indicated ETC subunits in wild-type and TAp73 KO cells, obtained using OXPHOS antibody cocktail. **I** qPCR against *mt-CO2*, expressed relative to expression of nuclear encoded *β2-microglobulin*. Relative expression was calculated using the ΔΔCt method and expressed as a percentage of wild-type control (n = 2 biological replicates, each tested in triplicate). n.s not significant in Student’s t-test.

Following the identification of an axis between TAp73 and OPA1, we investigated the morphology and function of the mitochondrial network in TAp73 KO cells. First, we addressed whether the observed OPA1 depletion was sufficient to impair mitochondrial fusion and alter steady-state mitochondrial dynamics. We visualised the mitochondrial network by immunofluorescence using an antibody to detect ATP5B. This revealed a striking fragmentation of the mitochondrial network in TAp73 KO cells compared with WT control cells (Fig. 2a). This was also recapitulated by siRNA knockdown of TAp73 (Fig. S3). Mitochondrial fragmentation was quantified in an unbiased assay, using the Intellesis trainable segmentation module (Zeiss), which quantified mean mitochondrial area across biological replicates (Fig. 2c). To confirm the observed mitochondrial phenotype was driven by TAp73 knockout, we performed a rescue experiment. The ectopic expression of TAp73 rescued a fused mitochondrial state. Likewise, mitochondrial fragmentation was rescued with ectopic OPA1 expression (Fig. 2a–c), placing OPA1 downstream of TAp73. We also observed perturbations in mitochondrial morphology in TEM micrographs of TAp73 KO cells (Fig. 2d, e). Overall, these data demonstrate a role for TAp73 in regulating mitochondrial morphology.

**Fig. 3:**
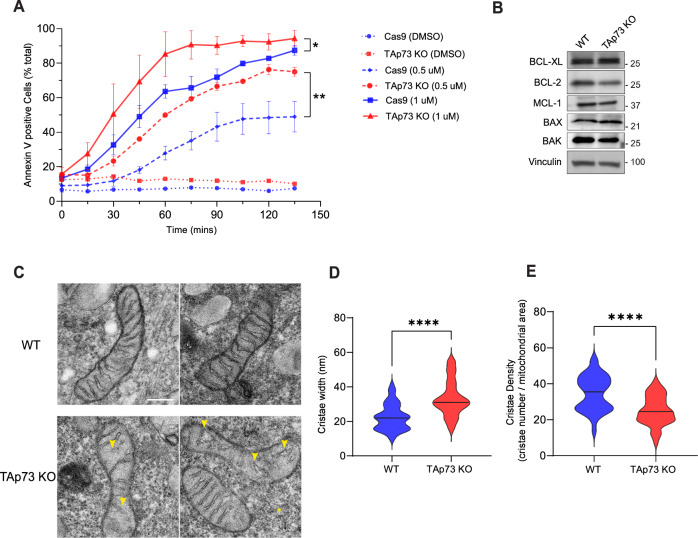
TAp73 KO cells are sensitised to apoptosis induced by BH3-mimetics. **A** Kinetics of apoptosis induction was tracked using AnnexinV/FITC dye following treatment with BH3-mimetic. Wild-type or TAp73 KO H1299 cells were treated with combination treatment of ABT-737 and S63845 at the indicated concentrations. Data points plotted as mean ± SD from n = 6 technical replicates. *P ≤ 0.05 (Student’s t-test), when comparing Cas9 control with TAp73 KO cells at 90 min and at the indicated dose of BH3-mimetic (0.5 µM and 1 µM). **B** Representative western blot against pro-apoptotic and anti-apoptotic proteins of the Bcl-2 family in WT and TAp73 KO cell lines. **C** Representative TEM micrographs of mitochondrial ultrastructure in WT and TAp73 KO cells. Yellow arrows highlight regions of disorganisation or loss of cristae in TAp73 KO cells. Scale bar = 200 nm. Quantification of mitochondrial cristae width (**D**) and cristae density (**E**) in TEM micrographs obtained from WT and TAp73 KO H1299 cells. Measurements were obtained from n ≥ 50 mitochondria, across two biological replicates. ****p < 0.0001 in Student’s t-test.

We next investigated the impact of TAp73 knockout on mitochondrial bioenergetics. Analysis using the Seahorse extracellular flux assay revealed that TAp73 knockout cells displayed a decrease in basal OCR, maximal OCR and spare capacity, which was rescued upon overexpression of OPA1 (Fig. 2f, g). These data therefore indicated that disruption of the TAp73/OPA1 axis elicited an impairment in electron transport chain (ETC) function, probably due to the role of OPA1 in maintaining cristae architecture and respiratory complex efficiency. In support of this concept, we found a disruption in cristae architecture in TAp73 KO cells (Fig. 3c–e). Moreover, we ruled-out that the depletion of mtDNA and/or a decrease in the protein level of ETC complex subunits was the mechanism driving the observed impairment in oxidative phosphorylation, as neither were significantly decreased (Fig. 2h, i).

### TAp73 knockout cells display an increased sensitivity to apoptosis induction with BH3-mimetics

The role of OPA1 in regulating mitochondrial fusion and cristae remodelling is linked with the execution phase of apoptosis. Upstream of caspase-9 activation, the mitochondrial inner membrane reshapes itself to facilitate the release of cytochrome *c* from the intermembrane space into the cytosol, where it binds to APAF1 and activates the assembly of the apoptosome [45]. The overexpression of OPA1 inhibits mitochondrial fission and apoptotic cristae remodelling, thereby rendering cells resistant to apoptosis induction by the intrinsic pathway [26].

To define the impact of altered mitochondrial morphogenesis on apoptosis induction in TAp73 KO cells, mitochondrial outer membrane permeabilization was triggered by treating cells with BH3-mimetics [46] (Fig. 3a). This approach allowed us to investigate a mitochondrial vulnerability in TAp73 KO cells whilst circumventing the role of TAp73 in orchestrating upstream modulators of apoptosis, such as PUMA and NOXA [47]. Due to the upregulation of multiple members of the pro-survival Bcl-2 family of proteins in H1299 cells, double agent treatment with ABT-737 (Bcl-2 and Bcl-xL inhibitor) and S63845 (Mcl-1 inhibitor) was necessary to induce apoptosis [48]. Quantification of the kinetics of apoptosis induction indicated that TAp73 KO cells were more sensitive to either 0.5 μM or 1 μM treatment of ABT-737 and S63845 in combination (Fig. 3a). Importantly, the observed sensitivity did not appear to be mainly driven by a downregulation of the pro-survival proteins targeted by BH3-mimetics (Fig. 3b). We additionally found that TAp73 knockout resulted in alterations in cristae architecture in untreated cells (Fig. 3c). The width of mitochondrial cristae was also significantly increased in KO cells, whilst the cristae density was profoundly decreased (Fig. 3e, d), consistent with a decrease in OPA1 expression.

### The TAp73/OPA1 axis is functionally relevant in the airway ciliated epithelium

The identification of a link between TAp73 and OPA1 regulating mitochondrial morphology prompted us to address the biological significance of this relationship in vivo. To do so, we analysed epithelial cells of the tracheal epithelium in *Trp73*^*−/−*^ mice, a tissue with high expression of TAp73 isoforms in mouse and human [49, 50]. In multiciliated cells (MCCs), TAp73 plays an important role by regulating the expression of key genes responsible for MCC function and homeostasis [19, 20].

**Fig. 4:**
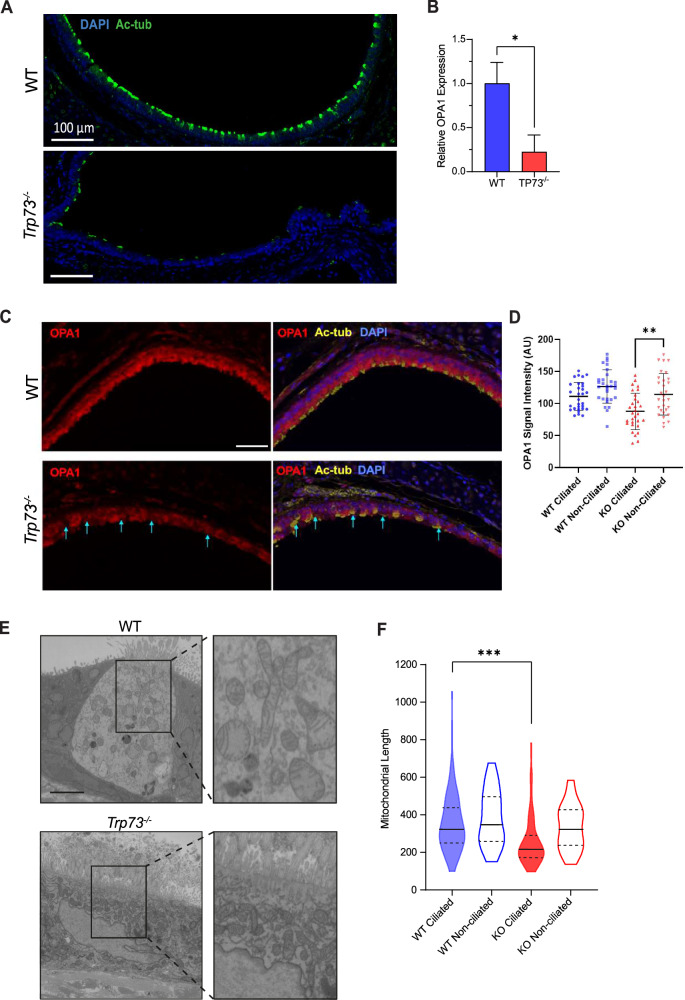
*Trp73*^*−/−*^ mice exhibit decreased OPA1 expression and altered mitochondrial dynamics in the airway ciliated epithelium. **A** Immunofluorescence staining performed against the cilia marker Ac-α-tubulin (green) with DAPI nuclear stain in tracheal cross-sections from WT and *Trp73*^*−/−*^ mice (blue). Scale bar = 100 μm. **B** RT-qPCR for *OPA1* mRNA expression in dissociated tracheal epithelial cells. Mouse trachea (n = 3 of each WT and *Trp73*^*−/−*^) were pooled together to obtain a sufficient number of cells. **C** Multiplexed IHC on mouse trachea against Ac-a-tubulin (yellow), OPA1 (red) and DAPI (blue). Cyan arrows indicate MCCs with low OPA1 expression. Scale bar = 30 μm. **D** Quantification of OPA1 expression in ciliated and non-ciliated cell populations from WT and *Trp73*^*−/−*^ mice. **E** Images of individual sections of MCCs from WT and *Trp73*^*−/−*^ ciliated epithelium obtained by SBF-SEM. Scale bar = 200 nm. **F** Mitochondrial length measurements were obtained from (E), including ciliated and non-ciliated cell populations. n ≥ 100 mitochondria from two samples of each genotype. ***P ≤ 0.001, n.s = not significant, in Student’s t-test.

We observed structural alterations in cilia in *Trp73* null mice (Fig. 4a), although the frequency of multiciliated cell loss was not as profound as previously reported [20], as 17% of cells successfully nucleated motile cilia; albeit with a defective architecture. This shows that TAp73 ablation manifests as a late-stage defect in the multiciliogenesis process, and was supported by the observation that TAp73 is not expressed in the progenitor cell compartment that expresses p63 (Fig. S4a).

We also performed immunofluorescence staining for forkhead box J1 (FOXJ1), a key transcription factor involved in motile ciliated cell differentiation which is positioned downstream of TAp73 to activate ciliogenesis genes [20]. This revealed the presence of FOXJ1 positive cells in the *Trp73*^*−/−*^ epithelium (Fig. S4b) and shows that additional molecular mechanisms - extending beyond TAp73 mediated fate determination and axonemal gene regulation - may underpin the multiciliated cell phenotype of *Trp73*^*−/−*^ mice.

Correspondingly, multiplexed immunohistochemical (IHC) staining of the *Trp73*^*−/−*^ tracheal epithelium showed a reduction in OPA1 expression (Fig. 4c, d). Moreover, co-staining for the ciliary marker Acetylated-α-tubulin revealed that OPA1 expression was reduced specifically in the ciliated cell lineage, from which *Trp73* was ablated (cyan arrows). Conversely, non-ciliated cells from *Trp73*^*−/−*^ animals displayed similar OPA1 expression to WT controls (Fig. 4d). Moreover, RT-qPCR indicated a reduction in OPA1 mRNA in the ciliated epithelium of *Trp73*^*−/−*^ mice, in support of a transcriptional regulation by p73 (Fig. 4b).

We next addressed whether decreased OPA1 expression in ciliated cells of the *Trp73*^*−/−*^ tracheal epithelium drives alterations in mitochondrial morphology by using serial-block-face scanning electron microscopy (SBF-SEM) to visualise the mitochondrial network. Individual sections from WT and Trp73^−/−^ ciliated epithelial cells (Fig. 4e) revealed a fragmented mitochondrial phenotype in knockout mice, which was confined to the MCC lineage (Fig. 4f), consistent with downregulated OPA1 expression. Furthermore, mitochondria from multiciliated cells were segmented in serial SEM sections to reconstruct the network in 3D (Fig. S5, Supplemental Movies 1, 2). The mitochondrial network in tracheal epithelial cells revealed a highly apical localisation of mitochondria in WT animals, consistent with previous reports [51]. This pattern of localisation was not observed in *Trp73*^*−/−*^ epithelial cells, suggesting an alteration in mitochondrial trafficking.

### *Trp73* and *OPA1* expression is dysregulated in COPD patients

**Fig. 5:**
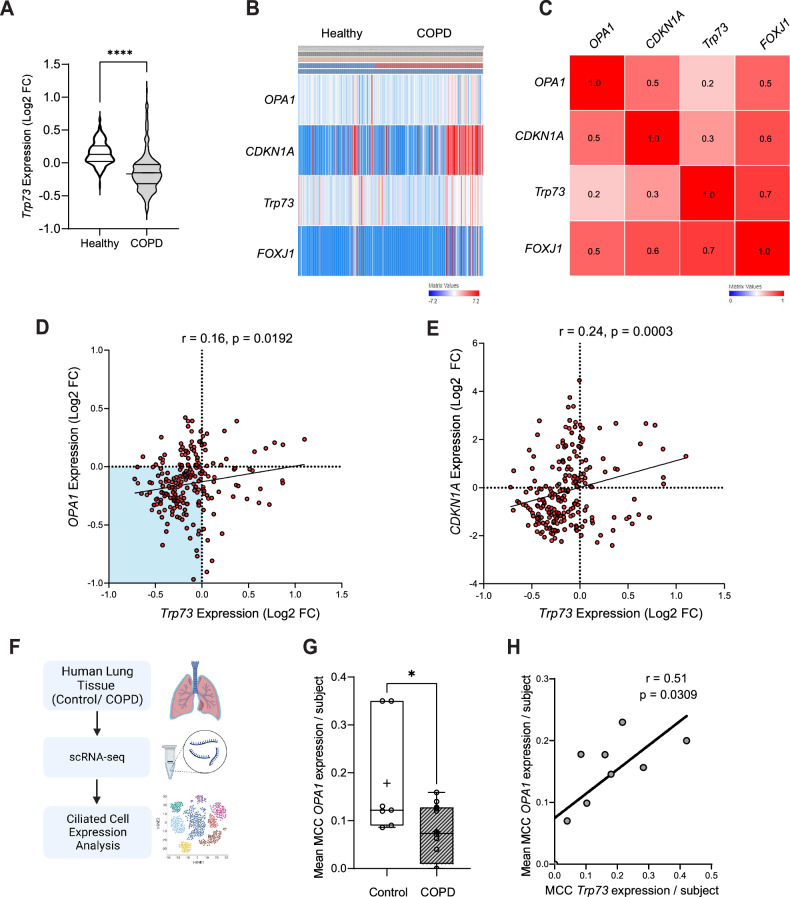
Expression of *Trp73* is decreased in COPD and correlates with *OPA1* expression levels. **A** *Trp73* expression data from healthy and COPD individuals from previously described Lung Genomics Research Consortium (LGRC) cohort (GSE47460; Affymetrix array data from n = 157 healthy and n = 220 COPD patients). **B** Heatmap of *Trp73, CDKN1A, OPA1* and *FOXJ1* expression in healthy and COPD individuals from the LGRC cohort. The heatmap was generated with the PulmonDB tool using Scipy library in Python, utilising cosine distance and average linkage. Red/blue cells represent positive/negative values. **C** Row similarity matrix indicating the association between each gene across patient data shown in (**B**). Red shading indicates a positive similarity (measured as 1 - cosine-distance, with similarity values indicated). **D**, **E** Expression data showing the correlation between *Trp73* and *OPA1* (**D**), or *Trp73* and *CDKN1A* (**E**) in COPD patients (GSE47460). Expression values are shown as Log2 fold change for the indicated genes relative to healthy control. The strength of the correlation was calculated using Pearson’s coefficient (r). **F**, **G** Analysis of scRNA-seq data from Control and COPD patients. Normalised gene counts were extracted for OPA1 in ciliated cell populations and displayed as mean expression per patient. Control n = 7, COPD n = 12, *P ≤ 0.05 in Student’s t-test. **H** Correlation plot of mean OPA1 and TP73 expression values per COPD patient. The indicated r value was calculated using Pearson’s coefficient.

**Fig. 6:**
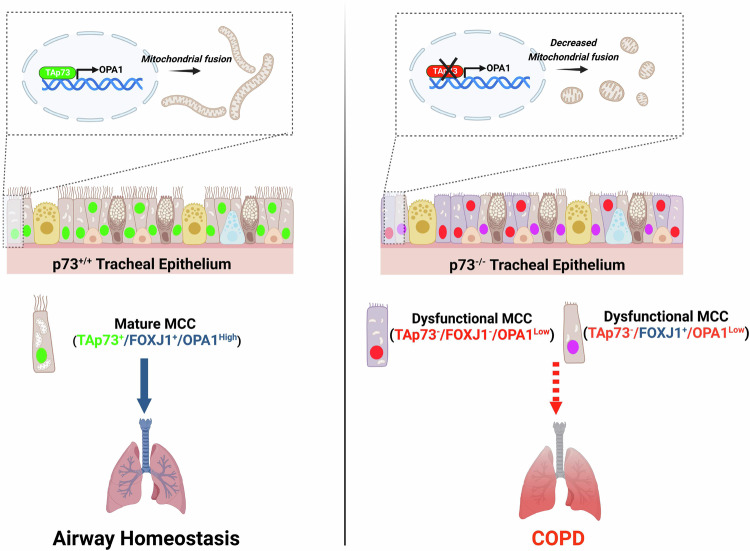
TAp73 regulates mitochondrial dynamics in vitro and in airway multiciliated cells in vivo. Schematic illustration showing the identified role of TAp73 in regulating mitochondrial dynamics. TAp73 expression is required for mitochondrial homeostasis in vitro and in the ciliated epithelium in vivo (green nuclei) (left). Conversely, TAp73 ablation leads to decreased OPA1 expression, mitochondrial fission, and impaired mitochondrial function (right). The *Trp73*^*−/−*^ tracheal epithelium maintains a limited expression of FOXJ1 positive cells (purple), indicating additional mechanisms, such as the observed mitochondrial dysfunction drives MCC loss and COPD pathogenesis.

Although Chronic Obstructive Pulmonary Disease (COPD) is characterised by a complex disease pathology and driven by multiple underlying mechanisms, it is often associated with multiciliated cell dysfunction, a loss or shortening of motile cilia, and reduced ciliary beating [52, 53]. Due to this similarity with p73 knockout mouse models, and the requirement for p73 for MCC function, we postulated that alterations in p73 expression could be implicated in COPD pathogenesis. Consistently, there was a highly significant reduction in *Trp73* expression in whole lung homogenates from COPD patients when compared with healthy controls (Lung Genomics Research Consortium; GSE47460) (Fig. 5a). Furthermore, we found a correlation between *Trp73* and *OPA1* expression across healthy and COPD lung cohort data (Fig. 5b, c). Indeed, extracting expression data from COPD patients highlighted a clustering of expression profiles with decreased expression of both *Trp73* and *OPA1*, driving a positive Pearson’s coefficient (Fig. 5d). However, a limitation of this bulk sequencing data set is the contribution of non-ciliated cell populations, demonstrated by the similar correlation coefficient obtained for *TP73*/*CDKN1A* expression, the latter being a known transcriptional target of TAp73 (Fig. 5e). We therefore reanalysed scRNA-seq data (GSE136831) obtained from lung explants of control and COPD subjects (Fig. 5f). Epithelial cells were clustered according to expression of canonical markers, as previously described [54], and expression data extracted for ciliated cell populations. This revealed that ciliated cell populations of COPD subjects had decreased expression of OPA1 relative to controls (Fig. 5g). We also found that TP73 expression correlated with OPA1 expression in FOXJ1 positive ciliated cell populations (Fig. 5f), affirming observations from bulk sequencing data. Overall, these findings highlight a dysregulation of p73 expression in COPD pathogenesis, and correlates with altered OPA1 expression, which was also observed in the *Trp73*^*−/−*^ mouse airway epithelium (Fig. 6).

## Discussion

We have identified a novel role for TAp73 in regulating OPA1 expression and thereby mitochondrial morphology and function. This finding has numerous implications, as the control of mitochondrial dynamics by the p53 family is a key mechanism for the regulation of cell metabolism (including metabolic shifts observed in cancers) [55], as well as the growth, dissemination [56], and differentiation [57] of tumour cells. Indeed, previous evidence has shown that p53 regulates mitochondrial morphology through the transcriptional regulation of MFN2 [58]. Nonetheless, a role of p73 in regulating fusion and fission genes has not been reported, and may underpin the diverse tissue dysfunction in p73 knockout mouse models [11].

The interrogation of in vitro and in vivo (mouse tracheal) ChIP-seq data sets [20, 44] revealed that TAp73 isoforms alpha and beta bind to overlapping genomic loci within the promoter region of numerous genes responsible for mitochondrial dynamics, which included OPA1 (Figs. 1 and S1). Moreover, highly similar binding patterns were observed for p53, indicative of a conserved ability of the p53 family to regulate mitochondrial shaping proteins. To this end, we performed targeted ChIP which confirmed the ability of TAp73 to bind the OPA1 promoter. The transcriptional regulation of OPA1 by p73 was also interrogated in TAp73^−/−^ H1299 cells, generated by CRISPR/Cas9 targeting (Fig. 1), and previously reported using an siRNA approach [9]. In a CRISPR KO model, the downregulated expression of OPA1 manifested as a profound alteration in mitochondrial morphology. Namely, TAp73 KO cells displayed a fragmented mitochondrial network, and crucially, this was rescued by ectopic expression of OPA1 (Fig. 2). However, the OPA1 rescue was not as effective as observed following transfection with TAp73 expression vector, suggesting the activity of additional regulators of fusion and fission may remain deficient. Together, this demonstrates a clear molecular dependence of the TAp73/OPA1 axis for the maintenance of mitochondrial dynamics (Fig. 2).

The alterations in OPA1 expression in a TAp73 KO model also demonstrate the importance of intricate control of mitochondrial shaping proteins for functionality of the organelle. OPA1 oligomers perform a myriad of functions that include, but are not limited to, fusion of mitochondria, the modulation of cristae morphogenesis, cytochrome c release, and respiratory complex efficiency [25, 59, 60]. The status of OPA1 as an effector of these mitochondrial functions therefore adds an additional layer by which TAp73 regulates mitochondrial bioenergetics, as we observed a defect in ETC function that was rescued with ectopic OPA1 expression (Fig. 2). Moreover, disruption of the TAp73/OPA1 axis appeared to increase sensitivity to apoptosis induced by BH3-mimetics (Fig. 3), possibly brought about by an increased propensity for cytochrome *c* release following cristae remodelling [26]. This finding is consistent with an upregulation of the p53 family and OPA1 in venetoclax resistant AML cells, and was unexpected due to the roles of the p53 family in inducing pro-apoptotic signalling pathways [47, 61].

The role of TAp73 in regulating mitochondrial dynamics may also extend to influencing broader aspects of cellular physiology and proliferation, as highlighted by the fact that following the ablation of TAp73, the growth rates of TAp73 KO cells were significantly impaired (Fig. S2c). Indeed, the positive regulation of OPA1 is broadly indicative of a growth promoting role of TAp73 isoforms, and should be addressed in further studies. This pro-growth function is consistent with a number of other roles of TAp73, such as the recently reported observation that it is required for cell cycle progression in H1299 cells [62], the regulation of glycolytic flux [15], and the regulation of ETC complexes [16]. Overall, these functions contrast with the over-simplified tumour suppressor dogma regarding TA-containing isoforms, and could explain why TAp73 expression is upregulated in many human cancers. Such a phenomenon may represent an out of context activation of the developmental roles of p73 [12].

In addition, we have demonstrated that OPA1 expression is downregulated in the ciliated tracheal epithelium of *Trp73*^*−/−*^ mice, revealing a relevance for the TAp73/OPA1 axis in vivo (Fig. 4). In line with this observation, alterations in mitochondrial morphology and ultrastructure were evident in the *Trp73*^*−/−*^ airway epithelium (Fig. 4; [9]). Moreover, the downregulation of OPA1 was confined to the ciliated cell lineage, specifically implicating TAp73 in modulating its expression (Fig. 4). Consequently, we propose that the disruption of mitochondrial function in ciliated epithelial cells may drive the phenotype of dysfunctional ciliogenesis, thereby causing COPD pathogenesis. Moreover, previous studies have shown that the pathogenesis of COPD is caused by alterations in mitochondrial dynamics [63]. However, functional assays rescuing MCC differentiation and function with OPA1 expression in the p73 null background are necessary to show that this axis is critical for multiciliated cell function.

It is also notable that *OPA1* heterozygous mice display abnormal brain development, thereby recapitulating features of neurological impairment observed following p73 loss [64, 65]. Inactivating *OPA1* mutations have been associated with human neurological disorders [66, 67], and is driven by an altered transcriptional circuitry in neural stem cells [68]. Given this striking similarity with the role of p73 as an essential regulator of neural stem cell maintenance in embryonic and adult neurogenesis [64, 69], future work may uncover a molecular relationship between TAp73 and OPA1 in neuronal cells or development of the nervous system. For example, the mechanism underpinning the loss of Cajal-Retzius neurons in the developing mouse hippocampus following oblation of TAp73α remains to be determined [10].

In summary, we have elucidated a novel transcriptional role for TAp73 in regulating mitochondrial dynamics. The regulation of OPA1 adds to the repertoire of p73 functions that are independent of the DNA-damage response. An understanding of such molecular mechanisms is important for paving the way for targeting p73 in conditions such as COPD, which is characterised by decreased p73 expression (Fig. 5). In addition to pathological conditions evident from p73 knockout mouse models, a relationship has been defined between cigarette smoke, decreased p73 expression and epithelial cell differentiation in vivo [41]. Taken alongside the role of TAp73 in regulating important ciliogenesis genes [19, 20], our findings show that alterations in the p73/OPA1 axis may represent a mechanistic link between MCC loss, chronic inflammation and airway disease (Figs. 5,6). Furthermore, TAp73-dependent regulation of mitochondrial morphology may also play a role in brain development and tumorigenesis.

## Methods

### Cell culture & transfection

NCI-H1299 cells (ATCC #CRL-5803) were cultured in modified RPMI 1640 (Gibco #A4736401, ThermoFisher, Waltham, MA, USA) containing 10% FBS (Gibco) at 37 °C and 5% CO_2_.

For transfection, cells were seeded at a density of 400,000 cells per well in 6 cm dishes and incubated overnight before transfection with Lipofectamine 2000 reagent (Invitrogen). Lipid-DNA complexes were formed in Opti-MEM medium (Gibco) before addition to cells. The media was replaced after 6 h and cells incubated for a further 24-42 h depending on the downstream application. Expression plasmid for OPA1 was purchased from Origene (#SC128155, Rockville, MD, USA), and HA-TAp73 cloned into the pcDNA3 backbone.

### TAp73 knockout generation using CRISPR/Cas9

H1299 cells were seeded at a density of 500,000 cells per 6 cm dish in 5 mL growth medium and incubated for 24 hours. Cells were transfected with 2 μg Cas9 expression plasmid (Horizon Discovery, Cambridge, UK) and 10 μL gRNA stock (2 μM; Custom Synthesis, Horizon Discovery; CAGGTGGAAGACGTCCATGCT). Transfection mixtures were prepared in 500 mL OPTI-MEM (Gibco) containing 5 μL lipofectamine before addition to cells. Cell culture media was replaced after 6 h and cells incubated for a further 18 h. Puromycin was added at a final concentration of 2.5 μg/μL and incubated for 16 hours. Clonal cell populations were obtained by dilution plating in 96-well plates. Once expanded, candidate clones were screened by western blot for TAp73.

Exon 2 spanning PCR fragments were amplified from gDNA of clonal CRISPR populations and cloned into the pJET1.2 blunt cloning vector using the CloneJET PCR cloning kit (Thermofisher, Waltham MA, USA). The ligation reaction was performed following the manufacturer’s instructions and using 1 μL of PCR product. NEB 5-alpha competent *Escherichia coli* (NEB #C2987H, Ipswich, MA, USA) were transformed with ligated plasmid by heat shock at 42 °C for 30 s. Cells were spread on pre-warmed LB Agar plates containing 100 μg/mL ampicillin (Sigma, St. Louis, MO, USA) and the plates incubated overnight at 37 °C. A minimum of 6 colonies were picked for overnight culture at 37 °C, shaking at 230 rpm. Plasmid DNA was obtained using the QIAprep Spin Miniprep Kit (Qiagen #27106, Hilden, Germany). Sanger DNA sequencing was performed by SourceBioscience (Cambridge, UK). Sequencing reads were aligned to the human TAp73 gene (GRCh38) using Geneious Prime software and INDELS identified.

### Animals

*Trp73*^*−/−*^ mice were previously generated in the BALB/c background [11]. All procedures followed guidelines and legislation as regulated under the Animal’s Scientific Procedures Act 1986 (ASPA) and were approved by the University of Leicester Animal Welfare and Ethical Review Body (AWERB). For all experiments excluding electron microscopy (EM), mice were euthanised by CO_2_ asphyxiation to preserve the trachea prior to dissection. For EM experiments, anaesthetised alexander mice were perfused via transcardiac perfusion.

### RT-qPCR

RNA was extracted from cell pellets using the RNeasy Plus mini kit (Qiagen #74136) according to the manufacturer’s instructions. For RT-qPCR of epithelial cells, dissected mouse tracheas were transferred to a 10 cm cell culture dish containing Ham’s F12 (Gibco) on ice. The trachea were cut open longitudinally to expose the inside surface. Three trachea from either wild-type or *Trp73*^*−/−*^ mice were pooled together in 15 mL falcon tubes containing 2 mL 0.15% filter sterilised Pronase (Roche, Basel, Switzerland) and incubated at 4 °C overnight. The tubes were gently inverted and FBS (Gibco) added at a final concentration of 10%. The tracheas were then transferred to a new 15 ml tube containing Ham’s F12/10% FBS and inverted. The contents from each tube were pooled and cells centrifuged at 300 × *g*, 4 °C for 10 min. The pellet resuspended in 600 μL DNase solution (Sigma; 0.5 mg/mL crude pancreatic DNase I, 10 mg/ml BSA, in Ham’s F12), and incubated on ice for 5 min. RNA was extracted using TRIzol reagent (Invitrogen), according to the manufacturer’s instructions.

Reverse transcription reaction was performed using RevertAid minus first strand cDNA synthesis kit (Thermofisher). Transcribed cDNA was diluted 2× before use in qPCR. Primer sequences (Sigma) are listed in Table 1. Reactions were carried out in triplicate using Fast SYBR Green PCR Master Mix (Thermofisher #4385612). The relative quantification was obtained using the Applied Biosystems 7500 thermocycler and quantitative comparative (ΔΔCt) method normalised to TBP.

**Table 1:** A list of primer sequences used for qPCR.

Gene	Forward primer (5′-3′) sequence	Reverse primer (5′-3′) sequence
**hTAp73**	CCAGACCTCTTCTTCCTC	GTCAAAGTAGGTGCTGTC
**mDNp73**	ATGCTTTACGTCGGTGACCC	GCACTGCTGAGCAAATTGAAC
**hp21**	CCTGTCACTGTCTTGTACCT	GCGTTTGGAGTGGTAGAAATCT
**hOPA1**	TCAAGAAAAACTTGATGCTTTCA	GCAGAGCTGATTATGAGTACGATT
**hTBP**	TCAACCCAGAATTGTTCTCCTTAT	CCTGAATCCCTTTAGAATAGGGTAGA
**hMFN2**	TCTCCCGGCCAAACATCTTC	ACCAGGAAGCTGGTACAACG
**hOPA1 promoter 1**	TCCATGCGCCATTGGGAG	CCTGCACTTACCAGGCCACA
**hOPA1 promoter 2**	ATGTAAGCCTCCCTCCCACT	TGTTACATGCCTAACCCACGAA
**hMDM2 promoter**	GGTTGACTCAGCTTTTCCTCTTG	GGAAAATGCATGGTTTAAATAGCC
**SAT2 promoter**	CTGCAATCATCCAATGGTCG	GATTCCATTCGGGTCCATTC
**Mt-Co2**	GCTGTCCCCACATTAGGCTT	ACCGTAGTATACCCCCGGTC
**β2-microglobulin**	TGCTGTCTCCATGTTTGATGTATCT	TCTCTGCTCCCCACCTCTAAGT

### Chromatin Immunoprecipitation

The MAGnify™ chromatin immunoprecipitation kit (Invitrogen) was used according to the manufacturer’s instructions. H1299 cells were transfected with TAp73α overexpression construct as described previously. Cells were fixed by adding PFA at a final concentration of 1% and incubating for 10 min at RT. Cells were lysed according to the manufacturer’s instructions and stored at −80 °C. Chromatin was sheared using the Covaris S220 set at a duty factor of 2%, intensity 4, and 200 cycles/burst for 6 min. Anti-HA antibody or mouse immunoglobulins was bound to Dynabeads® by rotating end-over-end at 4 °C for 1 h prior to IP. Chromatin was diluted to 200,000 cells per IP and immunoprecipitated according to the manufacturer’s instructions and eluted in 150 μL elution buffer.

### Western Blot Analysis

Cell pellets were lysed using RIPA buffer (Merck, Darmstadt, Germany) supplemented with 0.1% (v/v) protease and phosphatase inhibitor cocktail (Sigma-Aldrich, US). Protein concentration was measured using the Bio-Rad protein assay. Proteins were resolved by SDS-PAGE using 4–20% or 4–15% TRIS-glycine gradient midi gels (Bio-Rad, Hercules, CA, USA), in electrode buffer (0.1% w/v SDS, 192 mM glycine, 25 mM Tris; Bio-Rad) in BioRad criterion tanks. Protein was then transferred overnight onto a nitrocellulose membrane (Immobilon-P, Merk) using wet transfer for 16 h at 25 V in transfer buffer (25 mM Tris, 192 mM glycine and 20% methanol). The membranes were then blocked with 5% non-fat dry milk (Marvel) in TBST (0.1%) for 1 h at room temperature. The membrane was then probed with the appropriate primary antibody (Table 2) for 1 h at room temperature and washed with TBST. Membranes were then incubated with HRP conjugated secondary antibodies for 1 h at room temperature. Clarity Max Western ECL substrate was added to the membranes for 5 min (Bio-Rad #1705062) and chemiluminescent signal imaged using a ChemiDoc Imager (Bio-Rad). Quantification of Western blots was performed using ImageJ software. Pixel intensity of protein bands was normalised to loading control.

**Table 2:** A list of antibodies used in Western Blotting experiments.

Antibody target	Manufacturer	Catalogue number	Host species	Antibody dilution
TAp73	Bethyl	A300-126A	Rabbit	1:1000
OPA1	BD Biosciences	612606	Mouse	1:1000
Mitofusin-1	Cell Signalling	14739	Rabbit	1:1000
Mitofusin-2	Cell Signalling	9482	Rabbit	1:1000
p21	Santa Cruz	Sc-6246	Mouse	1:200
ATP5B	Abcam	Ab14730	Mouse	1:1000
GAPDH	Sigma-Aldrich	G8795	Mouse	1:5000
HSP-90	Santa-Cruz	Sc-13119	Mouse	1:1000
Human Total OXPHOS Cocktail	Abcam	Ab110411	Mouse	1:1000
MCL-1	Rockland	600-401-394	Rabbit	1:1000
BCL2	Santa-Cruz	Sc-509	Mouse	1:200
BCL-xL	Genetex	GTX100064	Rabbit	1:1000
Vinculin	Abcam	Ab18058	Mouse	1:5000

### Immunofluorescence

H1299 cells were seeded onto sterile glass coverslips (Type 1.5 thickness) and cultured overnight. Cells were fixed in 4% paraformaldehyde (VWR, Radnor, PA, USA) at room temperature for 20 min. The cells were washed 3 times in PBS and permeabilised at room temperature for 20 min in 0.2% Triton-X 100/PBS. Cells were washed with PBS/Tween (0.1%) 3 times, cover slips transferred to 12-well plates, and blocked in 5% Goat Serum/PBS/Tween for 1 h at room temperature. Anti-ATP5B antibody (mouse monoclonal, Abcam #ab14730, Cambridge, UK) was diluted 1:1000 in PBS and cover slips incubated overnight at 4 °C. Cells were washed 3 times with PBS/Tween (0.1%) and incubated with Alexa Fluor 488 goat anti-mouse secondary antibody diluted 1:1000 in PBS for 1 h at room temperature whilst protected from light. Cells transfected with HA-TAp73-pcDNA3 plasmid were incubated with rabbit anti-HA antibody (Santa Cruz #805, Dallas, TX, USA) diluted 1:200 in PBS for 1 h at room temperature, followed by detection with Alexa Fluor 568 goat anti-rabbit secondary antibody diluted 1:1000 in PBS. Coverslips were mounted on glass slides with Vectashield plus antifade mounting medium with DAPI (Vector Laboratories #H-2000, Newark, CA, USA). Images were acquired using a Zeiss LSM880 confocal microscope and 63x oil objective.

### Immunohistochemistry

IHC was carried out using the Ventana Discovery Ultra platform (Roche). Deparaffinization was performed by immersion in Ventana Discovery Wash buffer (Roche; 950-510) for 3 × 8 min washes at 69 °C. Antigen Retrieval was performed by incubation in Ventana CC1 buffer at 95 °C for 32 min. The slides were blocked by incubation with Discovery goat IgG block (Roche; 760-6008) for 12 min at 37 °C. Antibodies were diluted in EnVision Flex Antibody Diluent (Agilent; DM830, Santa Clara, CA, USA), at the dilutions indicated in Table 2. Multiplex antibody detection was performed using Opal fluorophores according to the manufacturer’s instructions (OPA1; Opal 690, Ac-α-tubulin; Opal 540; FOXJ1; Opal 570). Slides were imaged using the Ventana Discovery imaging system and quantification of signal intensity performed using In-Form software.

### Incucyte Annexin V/FITC imaging

H1299 cells were seeded at a density of 100,000 cells/well in a 12 well plate and cultured overnight at 37 °C and 5% CO_2_. Cell culture media was aspirated and fresh media added containing 1:3000 Annexin V/FITC dye (made in house by Dr Xiao-Ming Sun), 1 μM CaCl_2_, and incubated for a further 30 min. BH3-mimetics, ABT-737 (inhibitor of BCL-2 and BCL-xL) and S63845 (inhibitor of MCL-1), were added to cells at a range of concentrations ranging from 0.25 uM-10 μM per well or vehicle control (0.1% DMSO) and the cell culture plate placed in the IncuCyte live cell imager. Assay plates were scanned using the adherent cell-by-cell module and the 20× objective. Label-free counts were obtained using the phase contrast channel with a segmentation adjustment of 0.2 and a minimum area of 50 μm^2^. The green channel was imaged using an acquisition time of 300 ms and objects counted using the Top-Hat segmentation method with a threshold of 2.0 green calibrated units.

### Seahorse extracellular flux assay

Cells were cultured overnight at 37 °C, 5% CO_2_ in 96-well Seahorse microplates. Growth media was then removed, and cells washed three times in unbuffered DMEM Seahorse assay medium (32 mM NaCl, 2 mM GlutaMAX, 1 mM sodium pyruvate, 11 mM d-glucose, pH 7.4). The plate was then incubated in a 37 °C non-CO_2_ incubator for 1 h. The canonical mitochondrial toxins oligomycin A (port A), FCCP (port B), antimycin A and rotenone (port C) were added at the following final concentrations: 2 μM, 500 nM, and 2 μM, respectively, at 18 min intervals. Mean OCR values were obtained from a minimum of 5 wells per treatment and background subtracted. The data was normalised to cell number using Hoechst 33342 staining and individual wells were imaged using the 5× objective on a Zeiss Observer 7 microscope fitted with a Colibri 7 LED fluorescence light source.

### Conventional and large-volume electron microscopy

Mouse tissue sections and cells were fixed with half Karnovsky fixative as 2.5% glutaraldehyde and 2% paraformaldehyde in NaHCa buffer (0.1 dM NaCl, 30 mM HEPES, 2 mM CaCl2, pH 7.4) for 4 hours at room temperature. For conventional transmission electron microscopy (C-TEM) [70], post-fixation was performed with 0.25% osmium tetroxide + 0.25% potassium ferrocyanide and 1% tannic acid in 0.1 M sodium cacodylate buffer (pH 7.4). After staining *en bloc* with 5% aqueous uranyl acetate solution, the dehydration with a series of ethanol and the resin infiltration were completed for the plastic embedding in TER (TAAB Epoxy Resin). Ultrathin-sections (∼60 nm) were cut using an ultramicrotome (Leica EM UCT/UC7/Artos-3D, Austria), mounted in EM grids, and stained with lead citrate. Targets were observed using FEI Talos F200C (ThermoFisher) with Ceta-16M CMOS-based camera (4000 × 4000 pixels under 16 bit dynamic range) and JEM-1400 Flash TMP (JEOL Ltd., Japan) with TVIPS TemCam-XF416 CMOS (Tietz Video and Image Processing Systems GmbH, Germany) as described previously [9, 10].

For large-volume SBF-SEM [71], the tissues and cells were fixed with half Karnovsky’s fixative and post-fixed with osmium-thiocarbohydrazide-osmium (OTO) (1% osmium tetroxide + 1% potassium ferrocyanide in cacodylate buffer, aqueous 1% thiocarbohydrazide solution and aqueous 2% osmium tetroxide solution). *en-bloc* staining of fixed tissues was performed with aqueous 5% uranyl acetate solution. After dehydration and infiltration, samples were embedded in epoxy TER resin. Target regions were trimmed-down, mounted on aluminium pin stubs with conductive epoxy (CircuitWorks CW2400, Waukegan, IL, USA) and imaged by SBF-SEM in FEI Quanta FEG 250 (Thermo Fisher Scientific) equipped with ‘3View2XP’ system (Gatan Inc, Pleasanton, CA, USA) as described previously [72]. Back-scatter-electrons from the serial block-faces were recorded by Gatan ‘OnPoint’ detector at a pixel size 2.5 nm and beam acceleration of 3 kV under lower vacuum state of 40 Pascal, using the following parameters: spot size of 3.5, a dwell-time of 1.5 μs per pixel, ROI size of 10,000 × 25,000 (XY) pixels, 3 ROIs acquired for the montage, Z-slice thickness of 80 nm. 3D segmentation and reconstruction for the cellular tissue organelles were done by FEI Amira-3D, IMOD-4 (67), EMAN-2 [73].

### Human lung genomic data

Human lung bulk sequencing data was provided by the Lung Genomics Research Consortium (LGRC; http://lung-gemomics.org; 1RC2HL101715) using tissue samples and clinical data collected through the Lung Tissue Research Consortium (LTRC; http://www.ltrcpublic.com/), data series GSE47460. Gene expression analysis was performed using the PulmonDB tool [74].

Single cell RNA sequencing data of lung tissue previously published by Adams et al. was reanalysed focusing on individuals with and without COPD [75]. Clustering of cells was performed using the Seurat package (v.3.2.0 and v. 4.0.2.) in R, as previously described [54]. Expression values were normalised to 10,000 transcripts per cell and log-transformed using a pseudocount of 1. Mean expression values in ciliated cell populations were obtained from patients where >10 ciliated cells were recovered. Control and COPD samples were age-matched.

### ChIP-seq analysis

Publicly available ChIP-seq data sets (GEO series GSE15780) against p53 and p73 isoforms (TAp73α and TAp73β) in the human osteosarcoma cell line Saos-2 were interrogated for genes associated with the regulation of mitochondrial fusion and fission (39). BED files were converted to WIG files, and UCSC Genome Browser annotated tracks generated using the ChIP-Seq tools and web server [76]. ChIP-seq tracks were generated for each replicate and cross-checked against input tracks to account for non-specific signal. The maximum Y-axis scale was set to 100 for each genomic loci to allow for relative depiction of signal strength.

### Gene ontology analysis

GO analysis was performed on the complete set of genomic binding sites of p73 (1769 genes) identified In-Situ by Marshall et al. [20] using dissected murine trachea. The list of genes was analysed using the PANTHER GO tool v.17 overrepresentation test [77]. Genes were analysed against the complete set of Mus musculus genes annotated with the GO biological process complete annotation data set. Fold-enrichment values were plotted for selected GO classes (p ≤ 0.01).

## Supplementary information

Supplementary Figures 1-5 & Movies 1-2

Supplemental Movie 1

Supplemental Movie 2

ORIGINAL DATA

## Data Availability

All datasets on which the conclusions of this paper rely are presented in the main manuscript or additional supporting files.
