# Supplementary material for: TAp73 regulates mitochondrial dynamics and multiciliated cell homeostasis through an OPA1 axis
Source: Cell Death Dis. 2024 Nov 8;15(11):807. doi: 10.1038/s41419-024-07130-6 (PMC11549358; doi:10.1038/s41419-024-07130-6)
Supplement: Supplementary file 1 — Supplementary Figures 1-5 & Movies 1-2 [file 41419_2024_7130_MOESM1_ESM.docx]

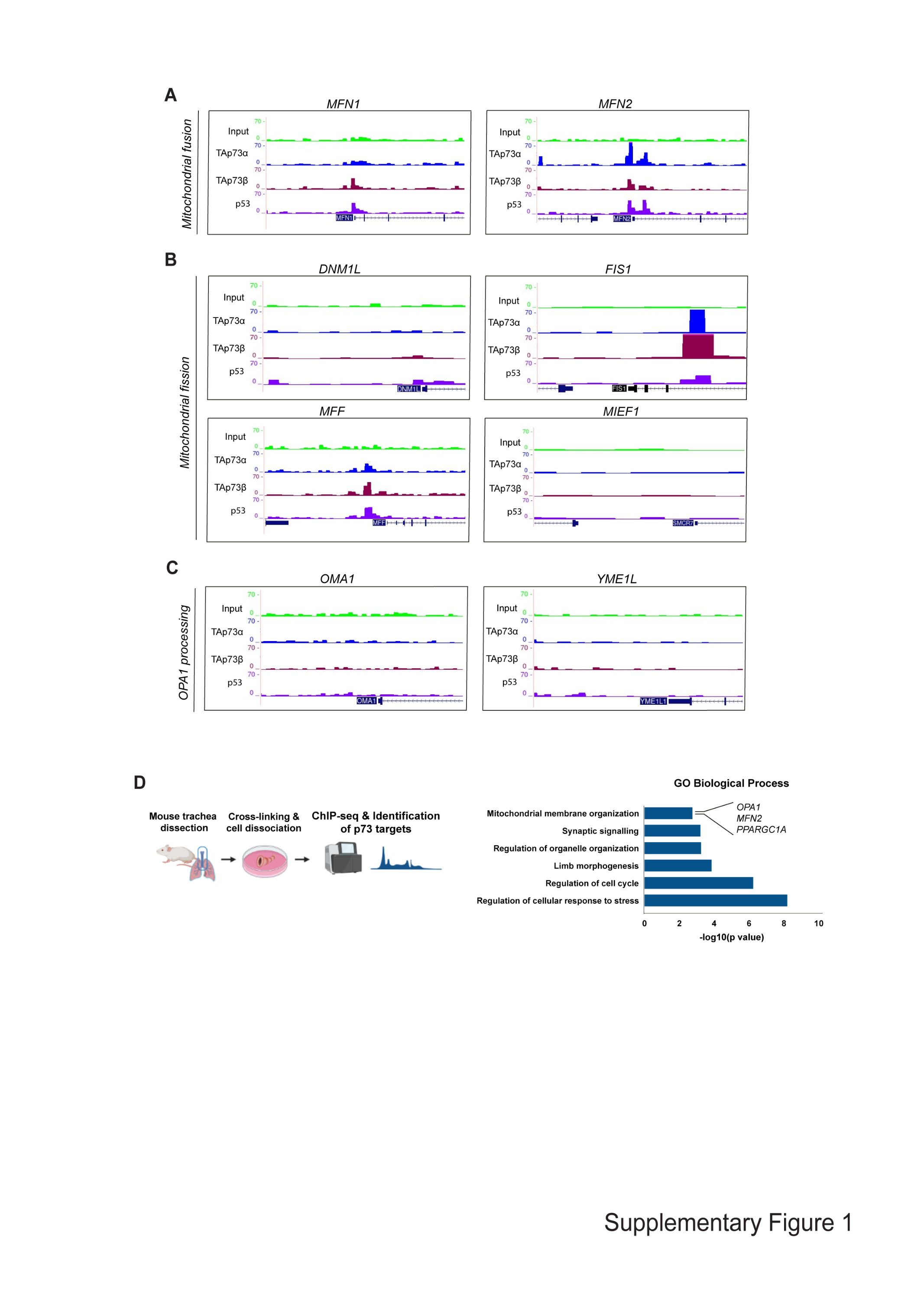


**Figure S1. ChIP-seq data shows TAp73 and p53 binding to a range of genes that regulate mitochondrial morphology.** (A-C) Interrogation of ChIP-seq data indicated binding of TAp73α, TAp73β and p53 to multiple regulators of mitochondrial fusion and fission. No TAp73 signal was evident for OPA1 processing factors (C). Input signal is shown in green. Sequencing read files were obtained from the GEO data set GSE15780. (D) GO enrichment analysis of 1769 TAp73 target genes identified using in-situ mouse tracheal ChIP-seq. Analysis was performed using the Panther GO overrepresentation test.


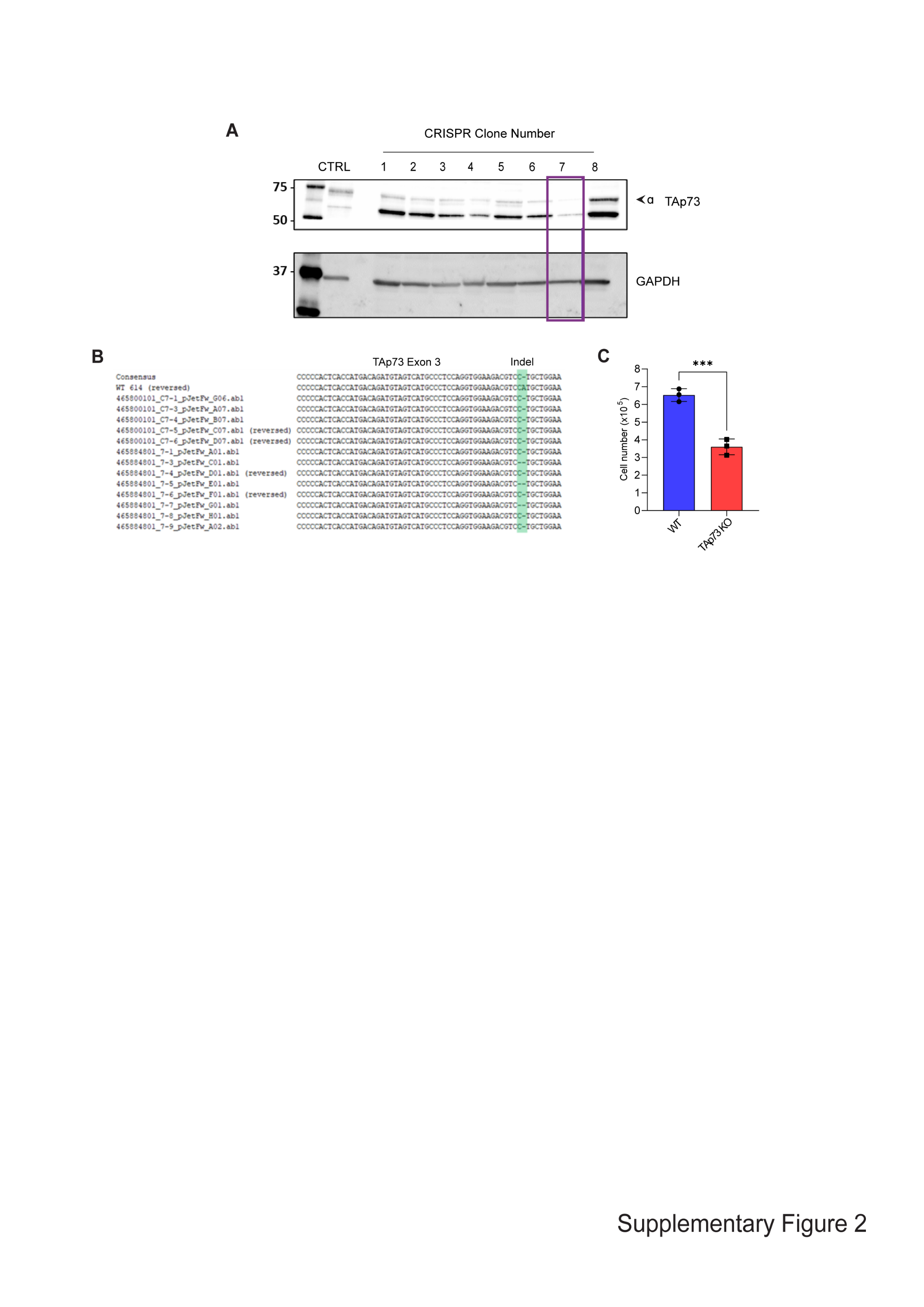


**Figure S2. Generation of TAp73 KO cell lines using CRISPR/Cas9 targeting.** (A) Representative western blot carried out to screen for TAp73 KO H1299 clonal populations. Clone 7 (purple box) was selected for Sanger Sequencing. (B) Sanger sequencing reads obtained from TAp73 KO CRISPR clone 7 aligned to the WT sequence. Primers were designed to amplify exon 3 of the Trp73 gene, encompassing the target site. The site of gene editing (INDEL) is shown in the green box at the DNA bases where CRISPR cells had a single or double base pair deletion immediately upstream of the PAM sequence. (C) Cells from the indicated cell lines were seeded at 20,000 cells/well in a 6 well plate and counted after 5 days to obtain relative rates of proliferation. Data are shown as mean ± SD (n=3). (***) P ≤ 0.001 (Student’s T-test).


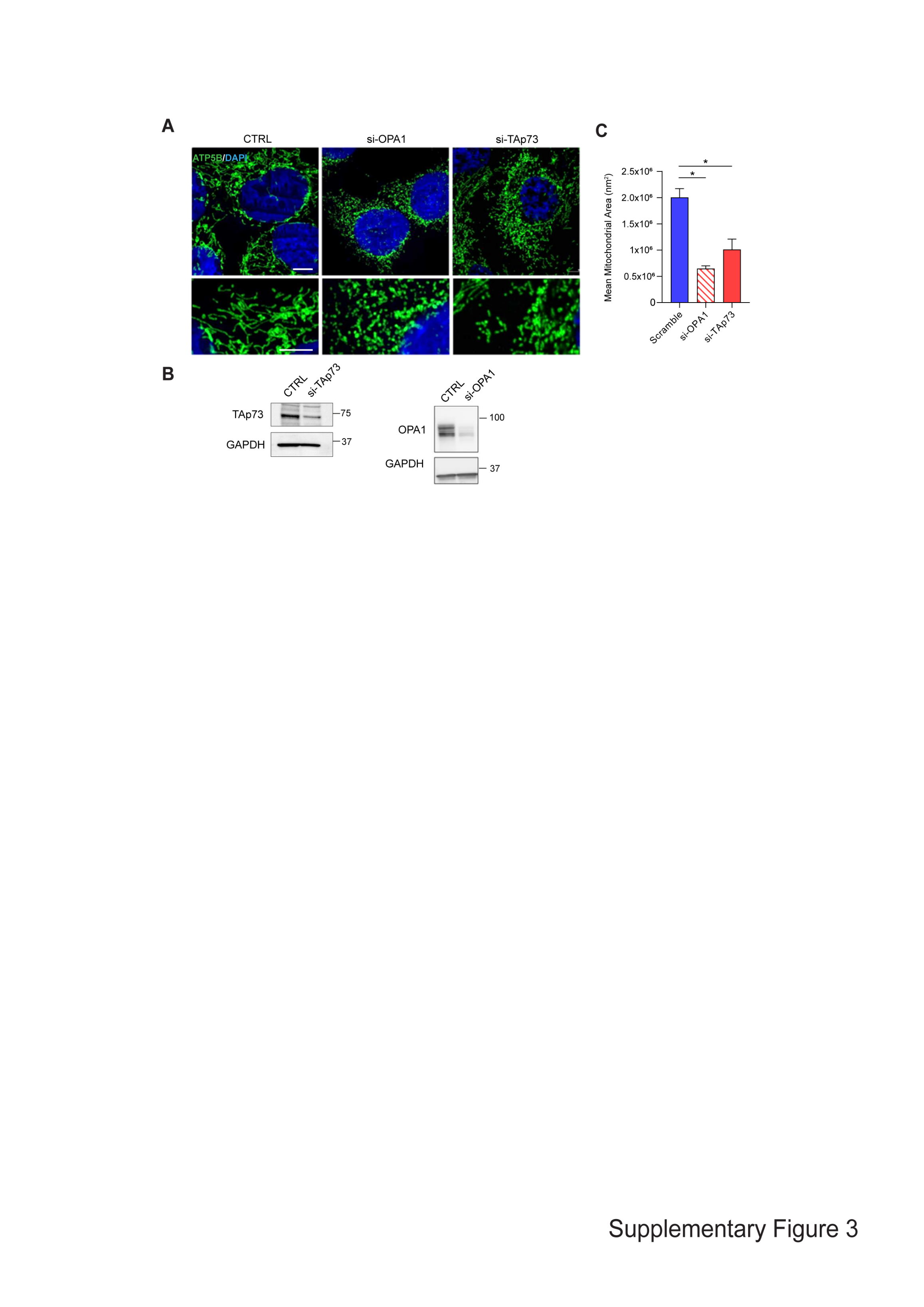


**Figure S3. H1299 cells treated with siRNA targeting TAp73 display fragmentation of the mitochondrial network.** (A) Knockdown of TAp73 was induced by siRNA. Cells were subsequently fixed and probed for ATP5B to visualise the mitochondrial network by immunofluorescence (green). The observed phenotype in TAp73 knockout cells is consistent with a depletion of OPA1 (middle panel). (B) siRNA mediated knockdown of TAp73 and OPA1 was confirmed by western blot. Images representative of two independent experiments (n=2). (C) Quantification of mitochondrial area in WT and si-TAp73 treated cells using Intellesis trainable object segmentation. (*) P ≤ 0.05 (Student’s T-test).


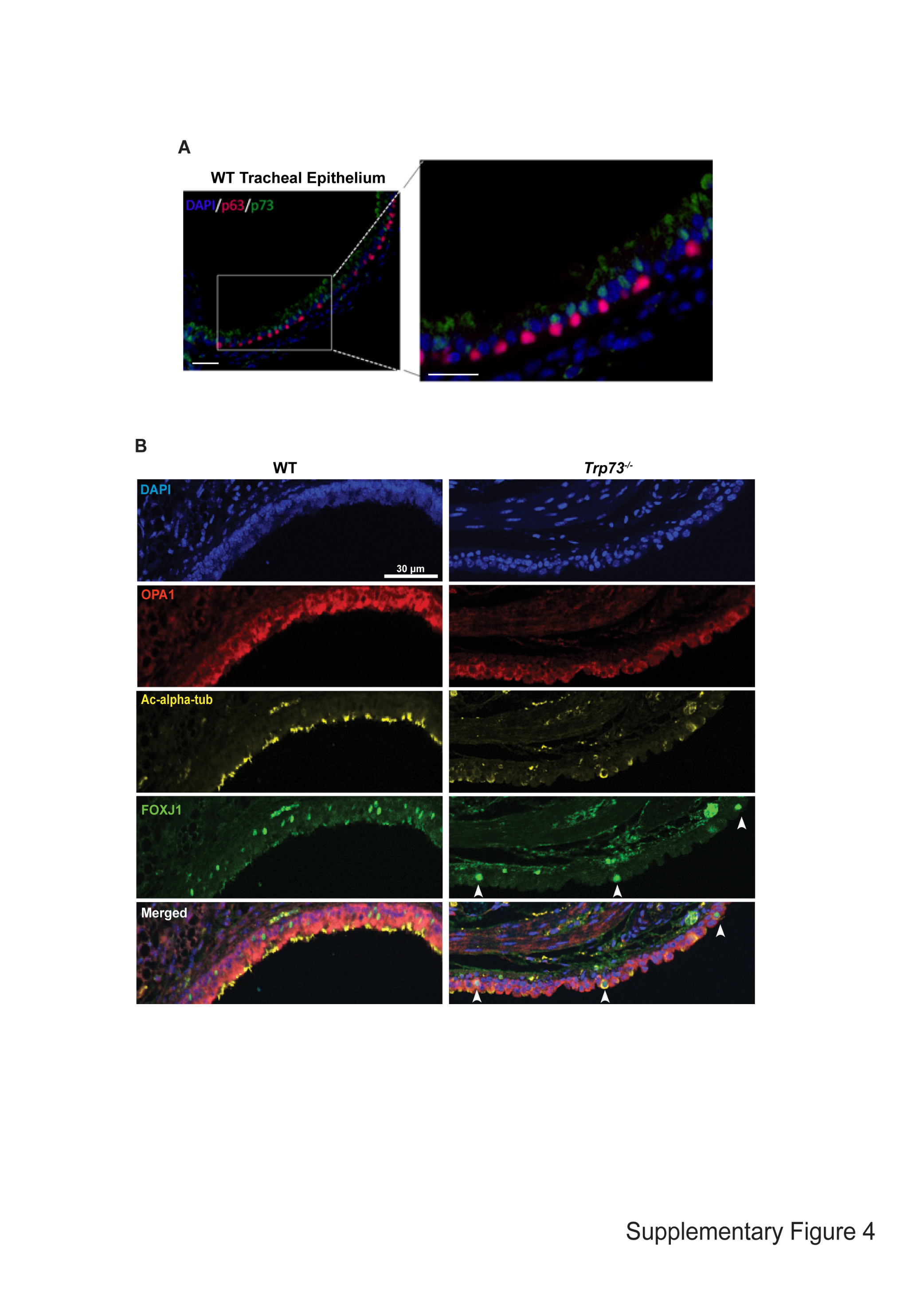


**Figure S4. TAp73 is not expressed in the basal cell compartment of the mouse tracheal epithelium, and the *Trp73*^-/-^ epithelium maintains FOXJ1 expression**. (A) Dual immunohistochemical staining for TAp73 (green) and p63 (pink) in the wild-type tracheal epithelium. No co-localisation of the signal was observed. (B) Single channel images from multiplex IHC staining performed in Figure 4. Staining for FOXJ1 was also incorporated. White arrowheads indicate FOXJ1 positive cell populations with perturbations in cilia structure.


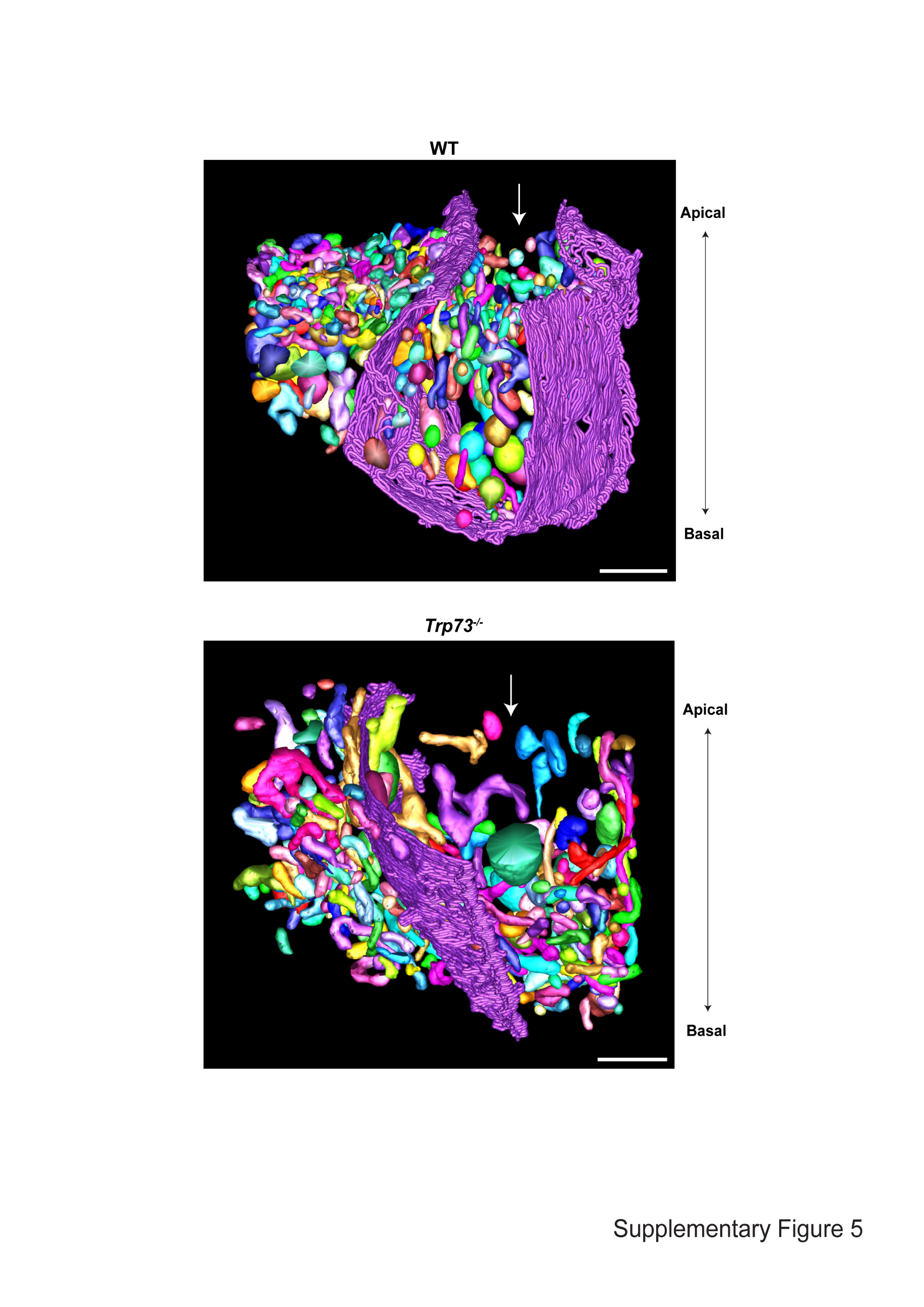


**Figure S5. SBF-SEM reconstruction of the mitochondrial network in ciliated cells of the tracheal epithelium.** Mitochondria were segmented from 100 sequential sections of tracheal epithelial cells (WT and *Trp73*^-/-^) to construct a 3D model of the mitochondrial network. White arrows denote ciliated epithelial cells, with cell boundaries indicated in purple. The apical and basal surfaces are indicated. Scale bar = 2500 nm.

**Supplemental Movie 1. Sequential sections from SBF-SEM micrographs from WT tracheal epithelium (Related to Figure 4E)**

**Supplemental Movie 2. Sequential sections from SBF-SEM micrographs from *Trp73*^-/-^ tracheal epithelium (Related to Figure 4E)**
