# Supplementary figures and images for: TAp73 regulates mitochondrial dynamics and multiciliated cell homeostasis through an OPA1 axis
Source: Cell Death Dis. 2024 Nov 8;15(11):807. doi: 10.1038/s41419-024-07130-6 (PMC11549358; doi:10.1038/s41419-024-07130-6)

FIG 1D

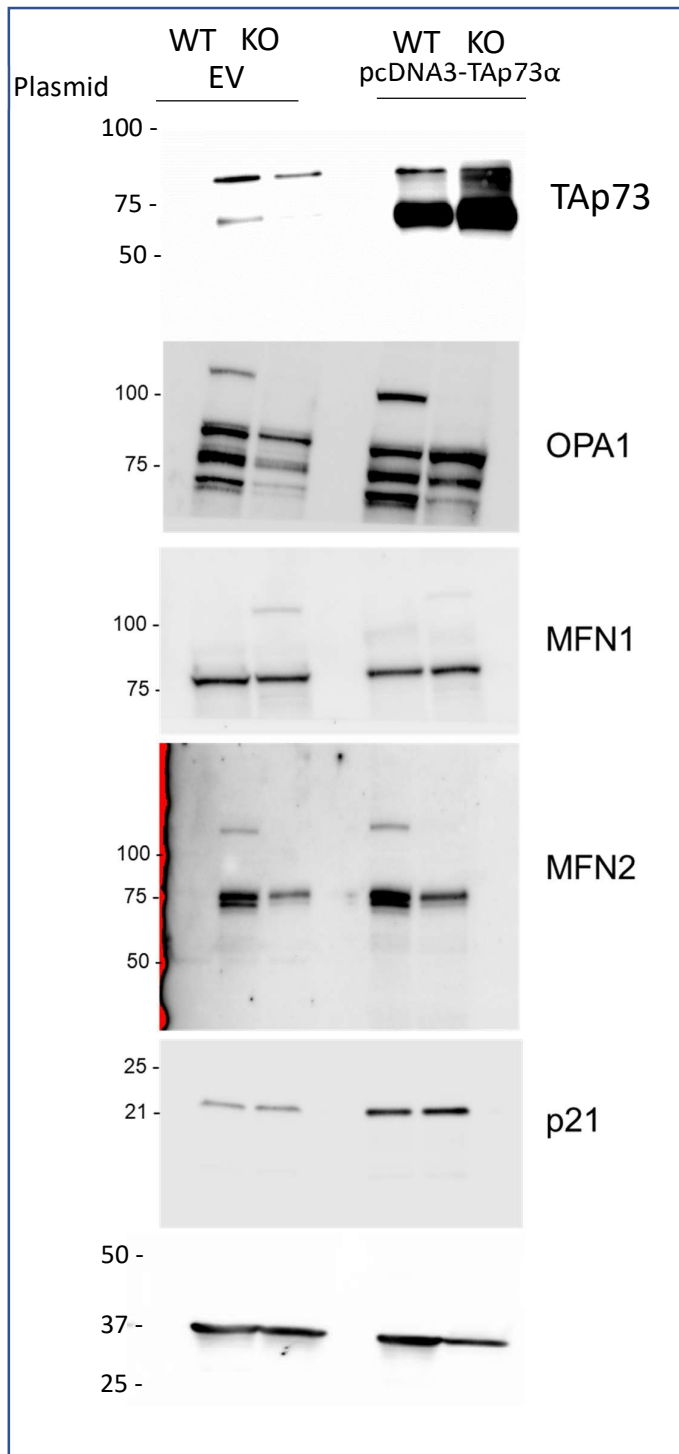

FIG 2B

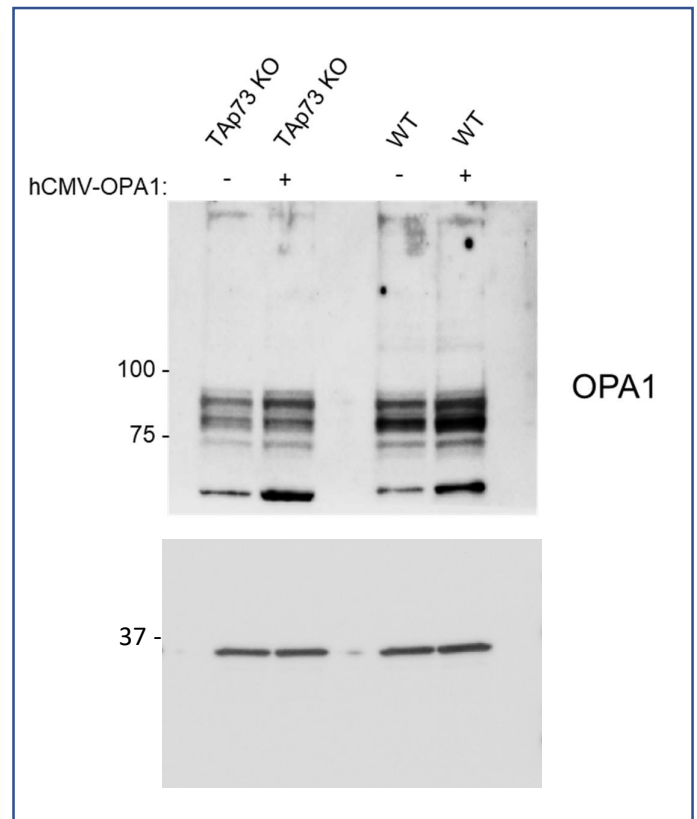

FIG 2H

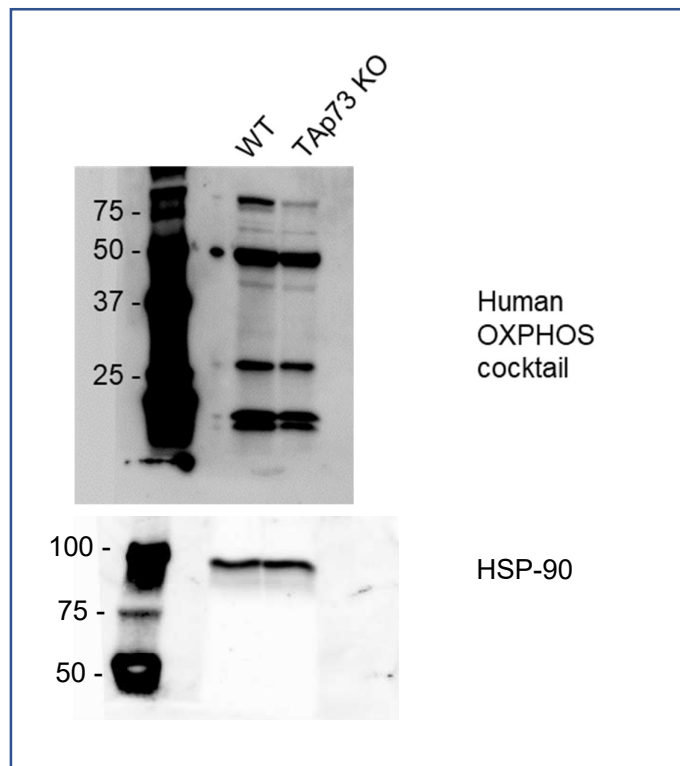

FIG 3B

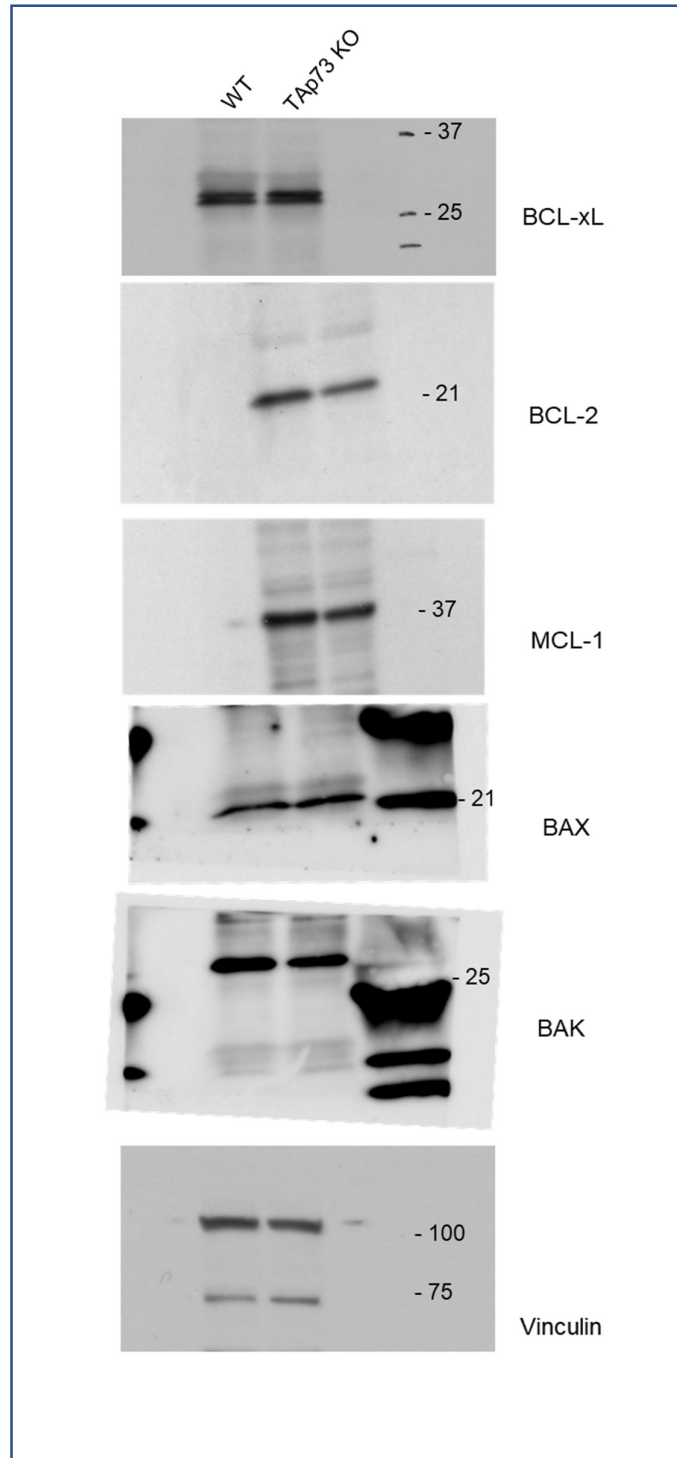

FIG S1A

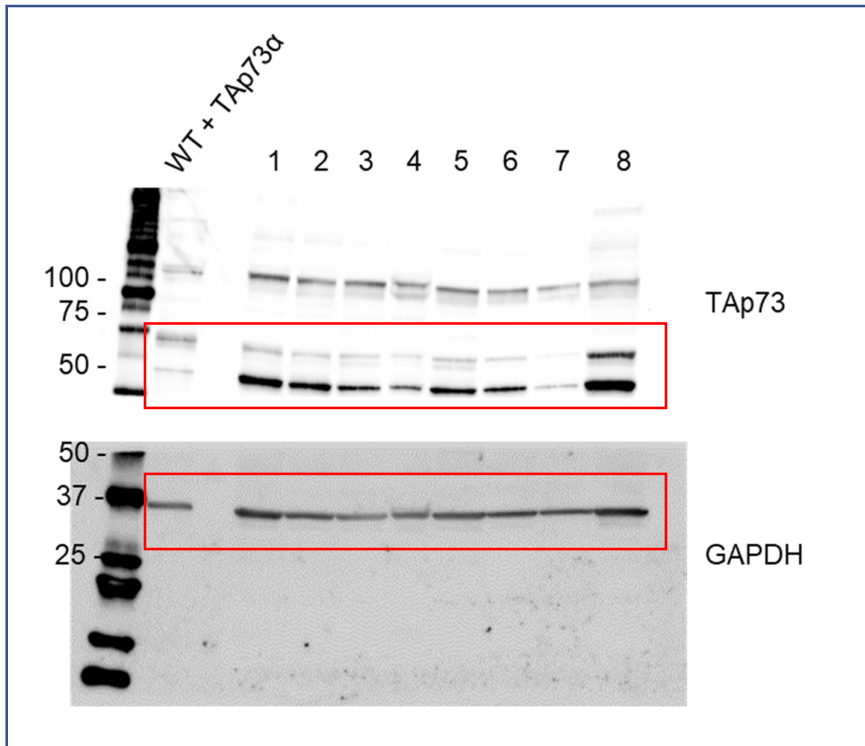

FIG S3B

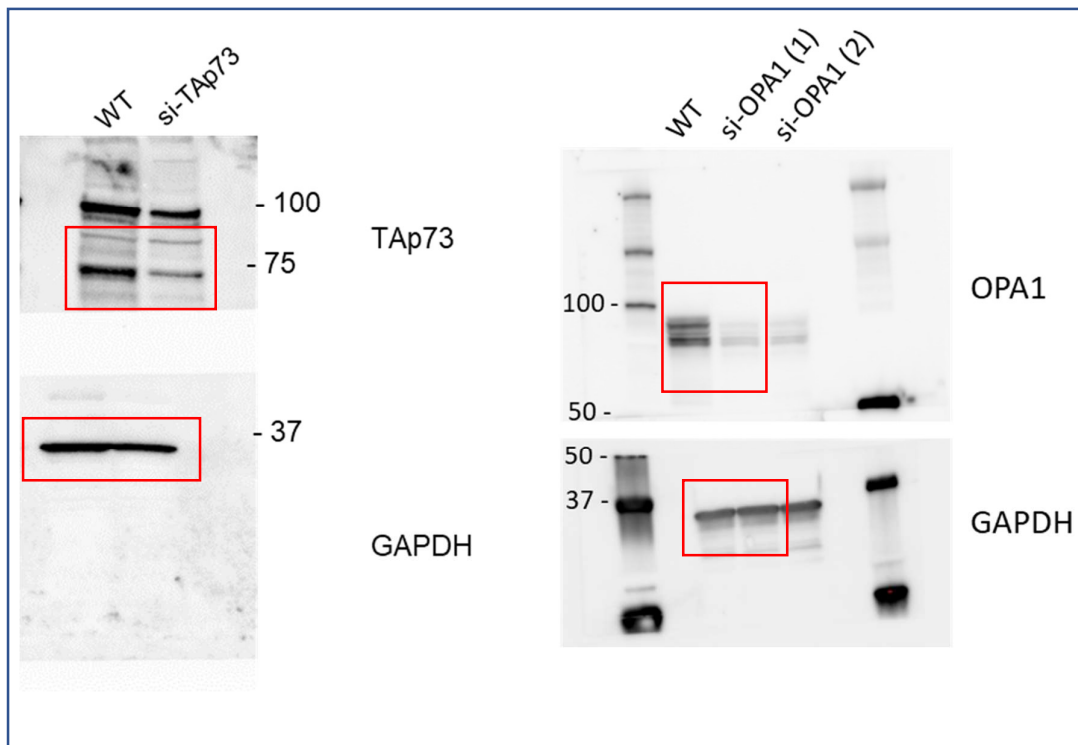

Supplement: Supplementary file 4 — ORIGINAL DATA [file 41419_2024_7130_MOESM4_ESM.pdf]
